# Percutaneous Vertebral Artery Access for Embolization of Cerebrovascular Disease: Illustrative Case and Operative Technique

**DOI:** 10.7759/cureus.1621

**Published:** 2017-08-28

**Authors:** Ali S Haider, Tijani Osumah, Richa Thakur, Steven Vayalumkal, Mrigank S Shail, Umair Khan, Hasan Sumdani, Joseph Hise, Kennith F Layton

**Affiliations:** 1 Texas A&M College of Medicine; 2 School of Medicine, Ross University; 3 School of Medicine, St. George's University; 4 School of Medicine, Xavier University School of Medicine; 5 Department of Radiology, Baylor University Medical Center

**Keywords:** arterio-venous malformation, aneurysms, coil embolization

## Abstract

Intracranial arteriovenous malformations can be further complicated by the development of aneurysms, which themselves carry the risk of rupture and hemorrhage. New endovascular techniques allow for more treatment options for these lesions in the setting of arteriovenous malformations. Here we present the case of a patient who developed an aneurysm in the setting of an arteriovenous malformation and subsequently underwent successful endovascular treatment via percutaneous access of the vertebral artery along with reviewing the literature on further treatment options and developments.

## Introduction

Intracranial arteriovenous malformations (AVMs) are rare vascular lesions associated with a risk of hemorrhage of up to 4% per year [1]. AVMs can be further complicated by the development of aneurysms in adjacent or feeder vessels that simultaneously carry a risk of rupture and hemorrhage. Flow-related aneurysms arise due to alterations in hemodynamic equilibrium in the AVM due to chronic high flow stress and endothelial damage [2]. Recent advancements in neuroendovascular surgery have broadened the treatment options for aneurysms and hemorrhage in the setting of AVMs, including detachable coil and liquid embolization agents as well as the use of microcatheters [1, 3-4]. Here, we present a rare and interesting case of flow related aneurysmal rupture in the setting of a tectal AVM requiring a percutaneous vertebral artery approach at the C1 level and detachable platinum coil embolization.

## Case presentation

**Figure 1:**
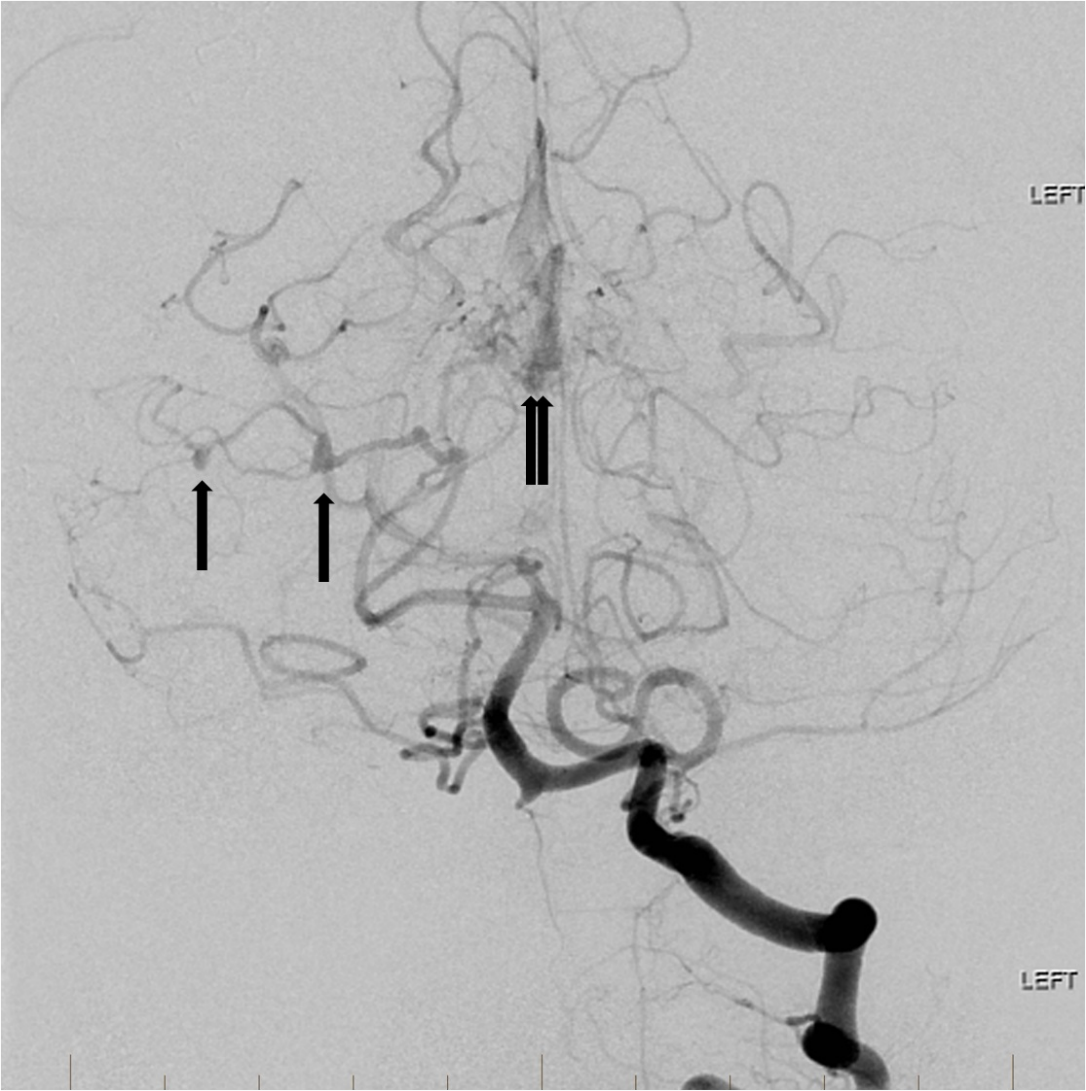
Left vertebral artery angiogram reveals a posterior fossa arteriovenous malformation (double arrow) with multiple dysplastic, fusiform aneurysms along the right superior cerebellar artery (single arrows). "Left" indicates the patient's left side.

A 63-year-old female with no significant past medical history presented to our facility with headache and neck stiffness. The headache progressed to being the most severe pain she had ever experienced. She was also mildly lethargic but did not exhibit any focal deficits. She denied history of trauma or use of aspirin or blood thinners. Initial brain computerized tomography (CT) demonstrated evidence of scattered subarachnoid hemorrhage and diffuse cerebral edema which was most prevalent within the posterior fossa. Subsequent brain magnetic resonance imaging (MRI) with and without contrast demonstrated persistent right superior cerebellar hemisphere subarachnoid hemorrhage with associated stable parenchymal hematoma and evidence of a distal flow-related right superior cerebellar artery aneurysm secondary to an underlying posterior fossa AVM. Cerebral angiography showed two ruptured flow-related aneurysms on an enlarged branch of the right superior cerebellar artery associated with a small tectal AVM (Figure 1).

**Figure 2:**
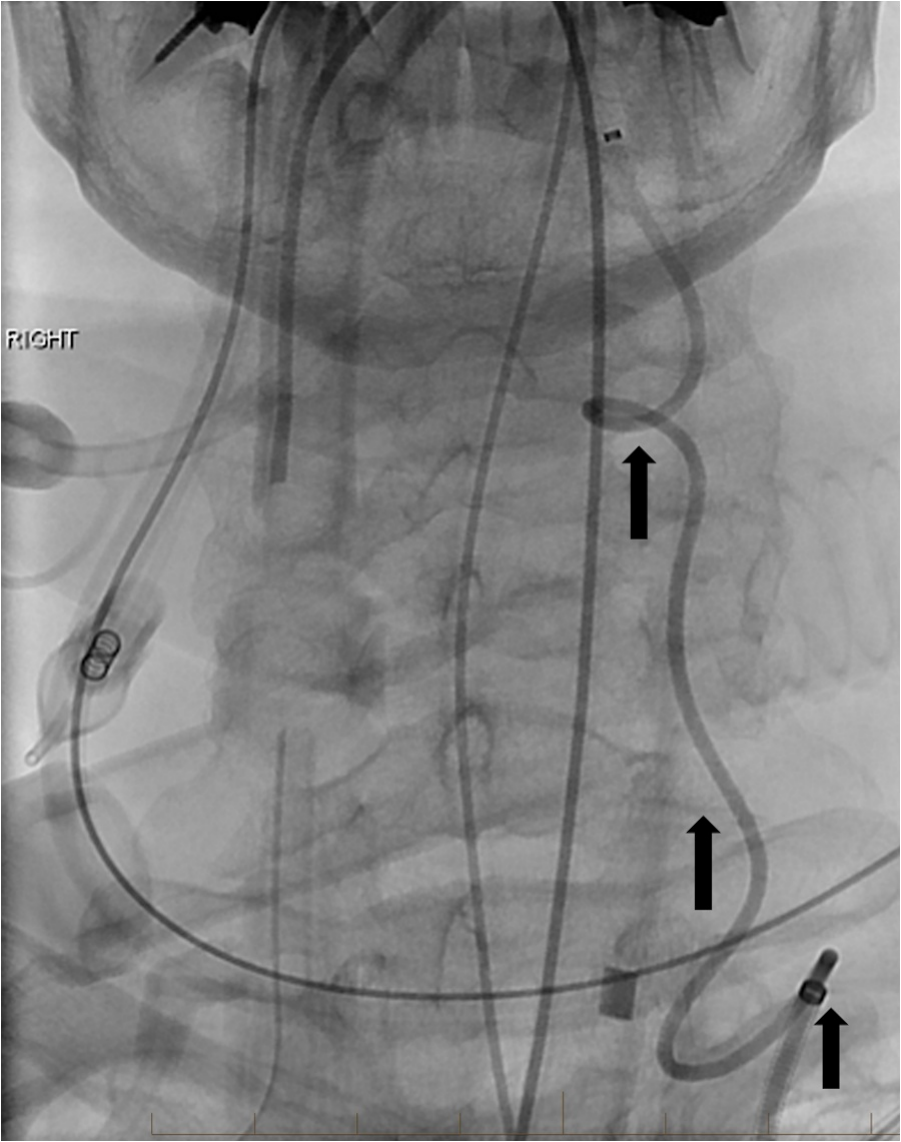
Frontal project fluoroscopic image reveals the markedly tortuous left vertebral artery (arrows). The tortuosity prevented adequate guiding catheter access to support the intracranial microcatheter manipulation. "Right" indicates the patient's right side.

However, initial attempts at endovascular treatment were unsuccessful due to profound tortuosity of the left vertebral artery, which was the only potential transfemoral or transradial access to the flow-related aneurysm (Figure 2).

**Figure 3:**
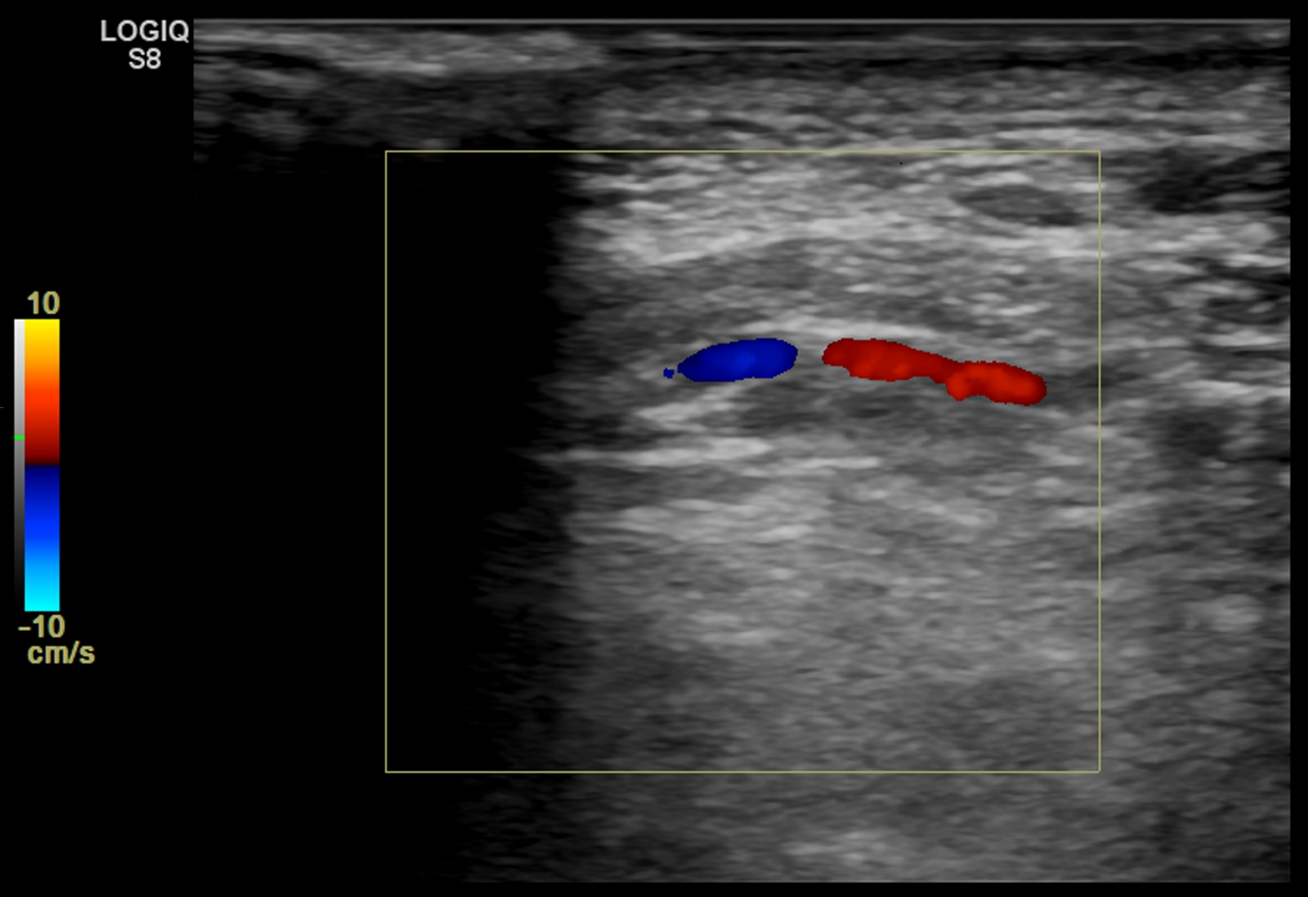
Duplex ultrasound image from the left lateral neck demonstrates the left vertebral artery near the C2 vertebra.

**Figure 4:**
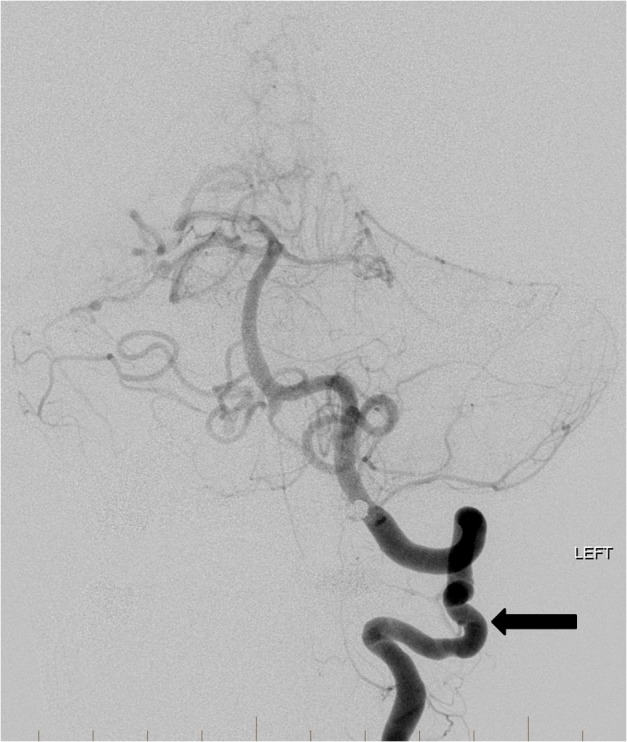
Frontal projection left vertebral artery angiogram was used as a roadmap for puncture of the vertebral artery (arrow) while simultaneously visualizing under ultrasound guidance. "Left" indicates the patient's left side.

Given the marked tortuosity of the vessels, the patient underwent treatment of the flow-related aneurysms beginning with percutaneous access of the vertebral artery at the C1 level, obtained using roadmap guidance from selective catheterization of the ostia of the left vertebral artery (Figure 3 and Figure 4).

**Figure 5:**
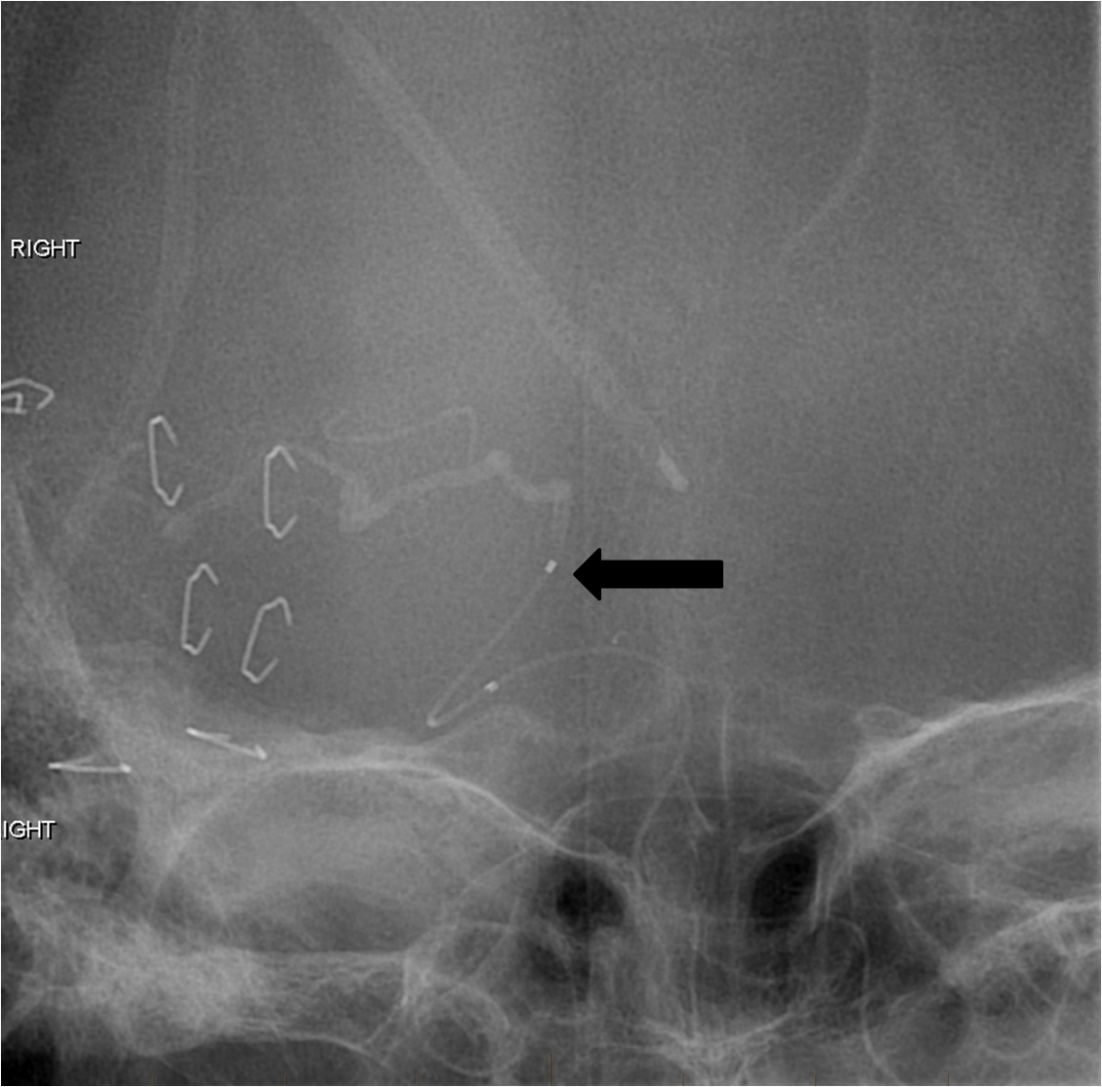
Frontal projection fluoroscopic image after successful left vertebral artery direct puncture. The microcatheter (arrow) has been advanced over a microwire into the right superior cerebellar artery harboring the dysplastic ruptured aneurysm(s). "Right" indicates the patient's right side.

Using a standard microcatheter system over a microcatheter guidewire, a super-selective catheterization of the two flow-related aneurysms was performed (Figure 5).

**Figure 6:**
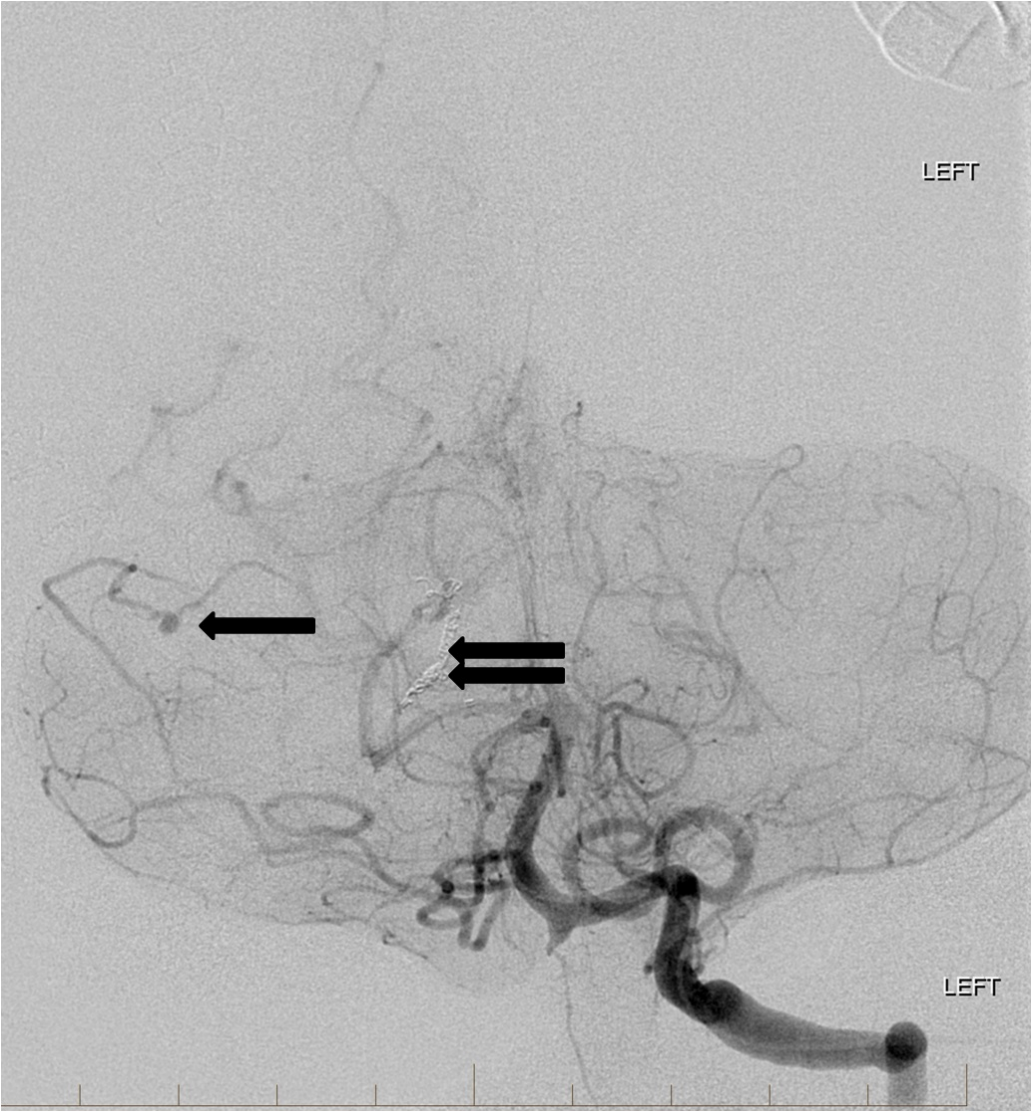
Following coil embolization (double arrow) of the right superior cerebellar artery branch harboring the dysplastic aneurysms, there is slow retrograde filling of the distal right superior cerebellar artery and aneurysms from right sided collaterals (arrow). "Left" indicates the patient's left side.

Multiple detachable platinum coils were then deposited into the aneurysmal branch resulting in occlusion of the vessel. Final control angiography revealed marked stasis in the two flow-related aneurysms well into the venous phase suggesting that they will rapidly thrombose (Figure 6).

**Figure 7:**
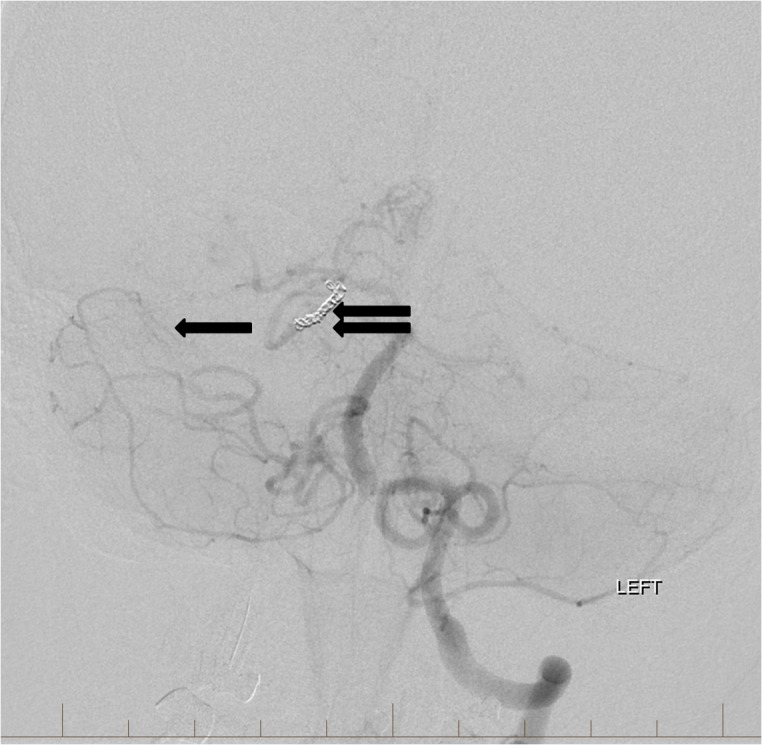
Follow-up left vertebral artery angiogram six months later demonstrates a stable position of the coils in the left superior cerebellar artery (double arrow). The distal left superior cerebellar artery dysplastic aneurysms are no longer visualized and are now completely occluded (arrow). "Left" indicates the patient's left side.

The patient subsequently underwent surgical resection of the AVM and made complete neurological recovery. Repeat angiographic evaluation at six months showed no evidence of vascular abnormality (Figure 7).

## Discussion

The incidence of AVM-associated aneurysms has been reported in the literature ranging from 2.7 to 58% with a prevalence of 10-20% being cited in the largest case series [1-2, 4-5]. It is thought that the altered hemodynamic stress and high-flow nature of AVMs cause endothelial damage and weakening of blood vessels over time, leading to aneurysm formation and increased propensity to rupture [2]. AVM aneurysm formation can be broken down further into unrelated, flow-related and intranidal of origin [4]. In a study by Redekop, et al., 29 (40.8%) of patients with AVM associated flow-related aneurysms presented with hemorrhage which was localized to the aneurysm itself in 12 (41.8%) of these patients [6]. Furthermore, recent reports show that flow-related aneurysms secondary to infratentorial AVMs are an independent predictor of poor outcome in patients [3,7]. This is in part due to the confined space and high concentration of neurological structures in the posterior fossa that are more likely to result in severe consequences due to hemorrhage or treatment complications [3]. Aneurysmal rupture in the setting of AVM and possible subarachnoid hemorrhage (SAH) is a feared complication associated with high morbidity and mortality, warranting emergent treatment when possible [1]. The mainstay of treatment for these aneurysms is a choice between microsurgical clipping of the aneurysm and in more recent years, endovascular treatment. Endovascular approaches include liquid embolization agents and flow-directed or flow-assisted microcatheters; these include various platinum coils as used on our patient and liquid agents such as Onyx and Histoacryl [1, 4]. High-flow aneurysms or aneurysmal ruptures may benefit from detachable coils for embolization, as used in our case with platinum coils, or selective flow reduction to avoid unwanted shunting through the venous system [4]. However, pre-existing hemodynamic derangements in AVMs can persist or worsen after embolization due to chronic loss of auto regulatory capacity in adjacent vessels and increased pressure on patent vessels. This could lead to disastrous re-hemorrhaging or subsequent aneurysm formation as detailed in a case by Gabrieli, et al. and Reynolds, et al. [5, 8]. Our case was also unique in that the tortuosity of upstream angioarchitecture required percutaneous access of the vertebral artery at the C1 level. This is in part due to undesired friction and rigidity within catheter advancement that impede placement of treatment devices in the cerebral vasculature. Dorfer, et al. outlined similar encounters with patients undergoing endovascular treatment that involved percutaneous puncture of the carotid or brachial arteries in order to gain safer access in an already high-risk procedure [9].

## Conclusions

AVM-associated aneurysms are a rare but dangerous complication of AVMs. The emergence of new endovascular techniques has allowed for broader management of AVM-associated aneurysms. Prompt treatment is necessary to prevent aneurysmal rupture, hemorrhage, and fatality. The patient we presented underwent successful endovascular treatment for an AVM-associated aneurysm using a novel approach. It is important for physicians to recognize and treat AVM-associated aneurysms promptly while taking into account the unique neurovascular anatomy for each patient in order to avoid potentially dangerous complications.
